# Eat whole and less often: ontogenetic shift reveals size specialization on kelp bass by the California moray eel, *Gymnothorax mordax*

**DOI:** 10.1007/s00442-018-4260-x

**Published:** 2018-09-18

**Authors:** Benjamin A. Higgins, Chris J. Law, Rita S. Mehta

**Affiliations:** Department of Ecology and Evolutionary Biology, Center for Coastal Biology, 130 McAllister Way, Santa Cruz, CA 95060 USA

**Keywords:** Dietary specialization, Kelp bass, Morays, Bite force, Predator–prey size relationships

## Abstract

Despite the importance of predation in many ecosystems, gaps remain in our understanding of nocturnal marine predators. Although the kelp forests of Southern California are some of the most well-studied ecosystems, California morays, *Gymnothorax mordax,* are predominately nocturnal predators that have remained largely unstudied and their predatory effects on the kelp forest ecosystem are unknown. We use a multi-year data set to examine the dietary breadth of *G. mordax* and to determine the functional role of this predator. We also quantify bite force to examine the potential performance limitations of morays in exploiting prey. Stomach content analyses and linear selectivity index values indicate that *G. mordax* specializes on kelp bass, *Paralabrax clathratus*. Average size of kelp bass consumed varies across years, suggesting that morays respond to fluctuations in prey size availability. The scaling relationship of kelp bass standard length and moray head length reveals an ontogenetic shift, where maximum prey size increases with moray size and small prey are dropped from the diet of larger individuals. Moray bite force exhibited strong positive allometry with moray head size, suggesting that larger morays exhibit greater bite forces for their head and body size. However, we found no relationship between prey size and bite force, suggesting that a disproportional increase in bite force does not facilitate the consumption of disproportionately larger prey. Our results indicate that while *G. mordax* of Catalina Island is a dietary specialist, it is capable of exhibiting functional shifts in prey size and species based on their abundance.

**Electronic supplementary material:**

The online version of this article (10.1007/s00442-018-4260-x) contains supplementary material, which is available to authorized users.

## Introduction

Predation dramatically affects the dynamics, relative abundance, and distribution of prey populations thereby influencing the pattern and direction of energy flow from lower to higher levels in food webs (Morin 2011). The effects predators have on their prey depends on the degree of prey specialization which is often dictated by a predator’s morphology (Werner 1977; Persson et al. 1996; Wainwright and Richard 1995) and whether predators themselves respond to changes in prey availability and density (Redpath and Thirgood 1999). Classification of predators as specialists or generalists informs the functional role of predators and how they may respond to fluxes in prey density (Andersson and Erlinge 1977). Whereas specialists tend to respond to changes in prey densities by immigrating to a new prey patch, generalists can respond similar to specialists or can respond functionally via prey switching (Murdoch 1969; Andersson and Erlinge 1977; Redpath and Thirgood 1999). Such frequency-dependent predation has shown to have a stabilizing influence on prey numbers (Redpath and Thirgood 1999) and maintain overall biodiversity in the ecosystem.

The terms ‘specialist ‘and ‘generalist’ also describe the breadth of the dietary niche. However, dietary niche is not only defined by the types of prey consumed but also by prey size. Prey size is particularly important when investigating how feeding behavior and diet may change over a species’ lifetime. There are a myriad of reasons as to why predators may become more successful at capturing prey at larger body sizes (i.e., increased muscle mass, larger gape, increased endurance). Therefore, it is expected that changes in body size may result in a linear shift in prey size (Arnold 1983; Persson 1990; Juanes 1994; Mittelbach and Persson 1998; Jacobson et al. 2018). Scharf et al. (2000) discovered that while larger predators (> 500 mm) tend to exhibit a narrowing in the breadth of relative prey sizes consumed over ontogeny (i.e., ontogenetic shift), asymmetric predator–prey size distributions appear to be a common pattern in aquatic communities (ontogenetic telescoping). These two patterns, ontogenetic shift and ontogenetic telescoping, which differ by whether small prey are dropped or maintained in the diet of large individuals, are important factors in determining predator–prey dynamics (Shurin et al. 2006) and provide a framework for understanding the mechanisms of the observed predator–prey relationships (Woodward et al. 2005; de Roos and Persson 2013).

Studying the ontogenetic changes to the underlying functional morphology of the feeding apparatus can also contribute insight into understanding predator–prey relationships. Functional measures of feeding performance such as bite force have potential to indicate resource use among the potential prey available (Osenberg and Mittelbach 1989; Pérez-Barbería and Gordon 1999; Marshall et al. 2012). Ontogenetic changes in bite force can facilitate specialization or generalization on different prey types and/or sizes and can, therefore, be used to elucidate asymmetries in resource use. For example, individuals that can exert greater bite forces can expand their dietary breadth by consuming larger or more robust food items (Verwaijen et al. 2002; Herrel et al. 2006; Bulté et al. 2008) and/or reducing handling times for both prey capture and consumption (Verwaijen et al. 2002; van der Meij and Bout 2006; Anderson et al. 2008). Thus, the maximum size of prey predators may be able to consume should change throughout ontogeny (Erickson et al. 2003; Sánchez-Hernandez et al. 2012). We expect that predator–prey size relationships will be especially strong in piscivorous predators that consume prey whole.

In this study, we examine the dietary ecology of the California moray eel, *Gymnothorax mordax.* A previous study revealed that morays, in general, exhibit specialized morphology for both the capture and transport of large prey (Mehta and Wainwright 2007). More recently, we have found that head length and vertical gape distance in the California moray increase disproportionately over ontogeny (Harrison et al. 2017), suggesting that the moray-feeding apparatus may be under strong selection to quickly increase gape size enabling larger individuals to consume larger prey items (Mittelbach 1981; Wainwright and Shaw 1999). Nevertheless, no study has recorded diet of the California moray in any detail and examined predator–prey size relationships.

Although researchers have traditionally identified California morays as predators in Southern California kelp forests with diverse prey handling strategies (Diluzio et al. 2017), their prey breadth remains unknown. The paucity of dietary information stems from the fact that California morays were thought to be relatively rare (Graham 2004; Froeschke et al. 2006). Recent work, however, revealed that the larvae of these cryptic predators are brought to Catalina Island during episodic El Niño events (Higgins et al. 2017) resulting in an abundant and relatively large biomass (~ 173.83 kg) of *G. mordax* within the rocky reefs of Two Harbors, Catalina Island (Higgins and Mehta 2017). As El Niño events have been shown to greatly alter the distribution of larval fish and resultant fish assemblages (Cowen 1985; Allen et al. 2002), one would anticipate temporal variation in predator–prey size relationships. Assessing temporal changes would require dietary analyses each year and over consecutive years, preferably incorporating dynamic climactic events such as an El Niño.

Here, we used a multi-year data set, incorporating the 2015 El Niño, to examine annual dietary patterns and to detect any size-based feeding habits of the California moray eel. Our objectives of this study were threefold. First, we examined the dietary breadth and determined, where the California moray fits on a continuum from generalist to specialist with respect to prey available in the environment. Second, we recorded in vivo bite force for a size range of morays to examine how feeding performance changes over ontogeny. Third, we use information on prey size and moray size to test whether average prey size increased over ontogeny. Through this multifaceted approach, we can better understand the trophic position and functional role of this elusive but abundant predator inhabiting the southern California kelp forest ecosystem.

## Materials and methods

### Trapping

**Fig. 1:**
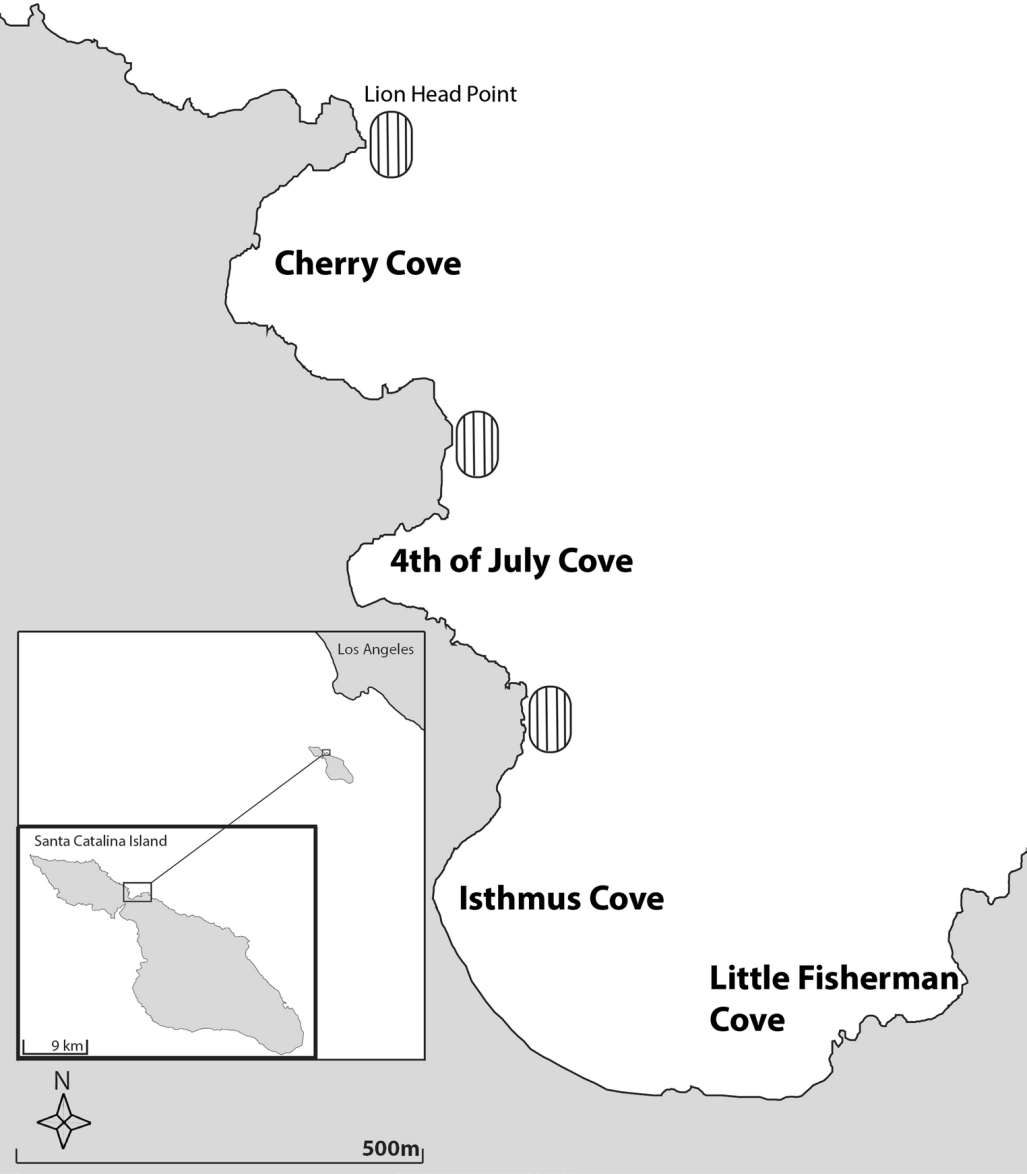
Map of two harbors, Catalina Island. Trapping locations are displayed in bold font. Ovals represent locations, where prey availability and abundance transects were conducted in 2013

*Gymnothorax mordax* were collected using custom-built, dual-chambered wire mesh traps (*N* = 20, 36″ × 11″ × 9″; Staten Island, NY) during the mid-late summer months (July–September) from 2012 to 2016 around Two Harbors, Catalina Island, CA (33°26′45.4″N, 118°29′31.3″W). Traps were set daily between 1800 and 1900 h and baited with frozen anchovies, which were placed into perforated plastic bottles allowing odor to serve as an attractant while prohibiting access to the bait. Mesh traps were randomly deployed within six trapping sites spread across four coves in Two Harbors (Fig. 1). Additional traps were set east of Lion’s Head Point (33°27′10.58″N, 118°30′3.94″W), and the slopes between Cherry and Fourth of July Coves (33°26′56.74″N, 118°29′57.49″), as well as Fourth of July and Isthmus coves (33°26′45.20″N, 118°29′52.44″W). These traps were used as the start/end points for prey availability and abundance transects. Traps were deployed around 1600 h and retrieved the following morning between 7:30 and 9:30 am.

### Gut content analysis and morphological measurements

**Fig. 2:**
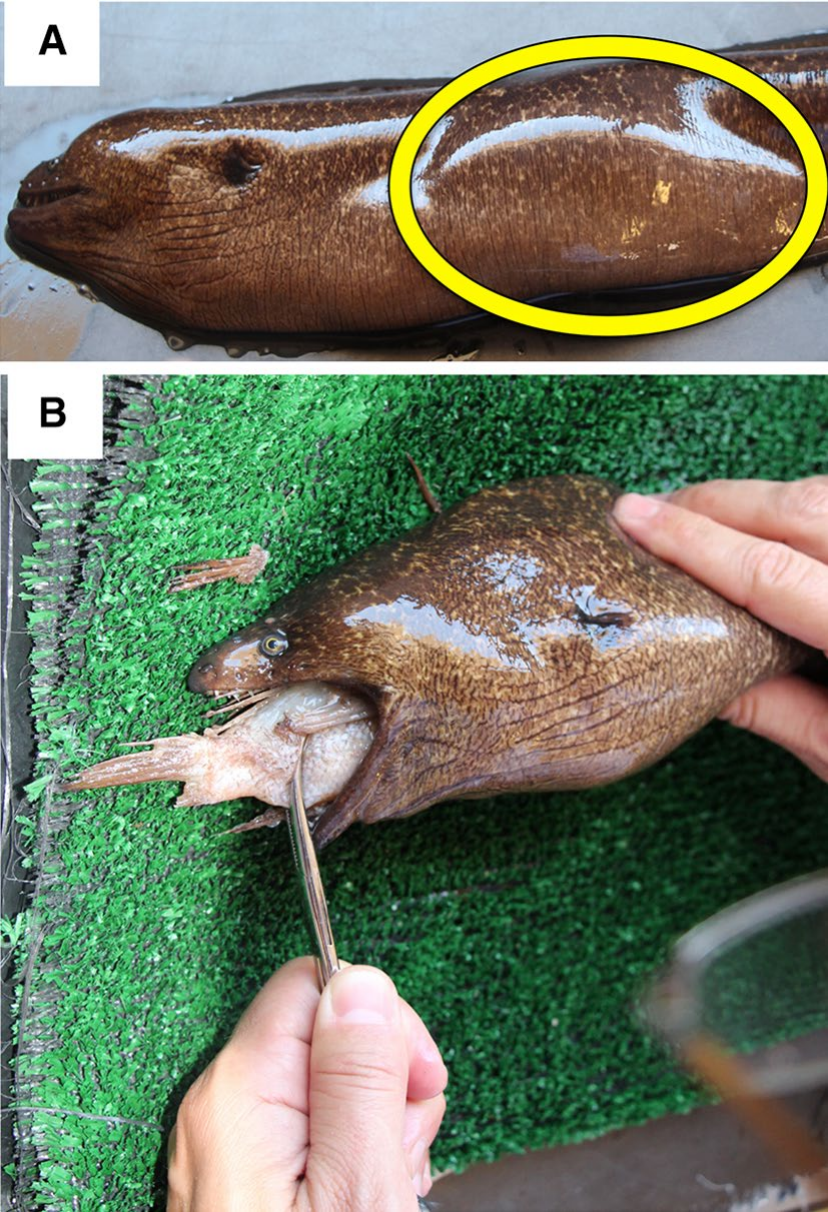
**a** Prey bolus indicated by circle on sedated *G. mordax.* **b** Example of how we extract a prey item in this case, kelp bass, using forceps. The item was first massaged towards the oral jaws via manual palpation

Trapped *G. mordax* were brought onboard a 6 m skiff. Individuals were placed in a lidded bucket filled with seawater and Tricaine Methanesulfonate (MS-222) buffered with sodium bicarbonate at roughly 90 mg/l. Once sedated, moray mass (g), total length (*L*_T_, mm; defined as distance from anterior tip of snout to posterior tip of tail), head length (*L*_H_, mm; defined as the linear distance between the anterior tip of the head and the posterior edge of the parabranchial opening), and head width (*L*_w_, mm defined as the linear distance spanning the lateral sides of the A2 subdivision of the adductor mandibulae muscles) were all recorded. Following these morphometric measurements, morays were examined for gut contents. We obtained consumed items via manual palpation, an effective and non-invasive method for recovering recently ingested items that is commonly used to obtain gut contents from snakes, another elongate predator that consumes prey whole (Mushinsky and Hebrard 1977; Fitch 1987). Prey items within the gut were massaged up from the bottom of the stomach and into the mouth from where they were carefully extracted with forceps (Fig. 2). All dietary items recovered were identified to lowest taxonomic group, and maximum lengths (mm) for whole prey were recorded.

### Prey availability and abundance

Observations of potential invertebrate and vertebrate prey were obtained from Reef Check California’s online Global Reef Tracker database. These data enabled us to analyze prey availability and abundance for all years included in the present study (2012–2016). Reef Check California surveys the relative abundance and size distribution of species using a methodology based on the Department of Fish and Game’s Cooperative Research and Assessment of Nearshore Ecosystems (CRANE) monitoring program. Transects are conducted in both onshore and inshore reefs with a maximum depth limit of 18 m, with a series of three transects spanning 30 m each. Transects are conducted annually at the same sites in which morays were collected. In 2013, we conducted our own prey availability and abundance transects to corroborate the Reef Check survey results and found strong correspondence (see supplemental materials).

To categorize morays as predators on a continuum from generalist to specialist, we used the linear selectivity index (*L*; Strauss 1979), which requires knowledge of both the prey items available in the environment and those found through gut content analyses. *L* selectivity is a unitless number and is the unweighted difference of the proportions of prey items found in the gut and the same item(s) recorded in the habitat. Thus, an *L* value of 0 would indicate that *G. mordax* is not specializing on any particular prey item(s) and is a generalist consumer, whereas a value closer to 1 suggests that there is strong specialization for a particular prey, while negative *L* values indicate avoidance, or inaccessibility of prey.

### Bite force

To quantify moray-feeding performance over ontogeny, we measured in vivo bite force for as many of the trapped individuals as possible. We used a piezoelectric force transducer (Kistler Quartz Force Sensor type 9203) mounted between custom-made steel cantilever beams and fitted with a handheld charge amplifier (Kistler type 5995A). Steel bite plates were fixed onto the cantilever beams and set 2.4 cm apart. Following recommendations from Lappin and Jones (2014), we covered the steel bite plates with leather to reduce stiffness of the bite plates and to avoid subjecting morays to possible tooth and jaw damage during biting trials. We recorded maximum bite forces from trapped individuals. An individual moray was placed in a 5 gallon bucket to constrain the body of the animal and the force transducer was positioned in front of the moray’s mouth to elicit biting. All bite force data were recorded from anterior bites. Therefore, morays presumably bit with the peripheral and median intermaxillary teeth and teeth along the anterior dentary of the oral jaws. We then sedated each individual to record the same suite of morphometric data (mass, total length, head length, and head width) as described above, and then released individuals to their original coves, once they recovered from anesthesia. These data were collected during the 2015 and 2016 summer months.

### Statistical analyses

All statistics were carried out in R 3.4.1 (R Core Team 2017). For each year, we tallied the number of prey items and taxonomically grouped items (e.g., fishes, crustaceans, and mollusks) found in the moray diet. We tested whether the number of prey items consumed and whether the proportions of different types of prey items consumed varied between years using Kolmogrov–Smirnov tests. Repeated Hotelling’s two-sample *t* tests were used to determine if consumed prey proportions differed across years (R package “Hotelling”). For each year, we also presented the size distribution of kelp bass prey, because we found kelp bass to be the dominant prey for morays (see Results). We tested for differences in the sizes of kelp bass consumed across years using an ANOVA followed by a Tukey’s honest significant test (HSD) to examine pairwise differences across years. The variances of the annual kelp bass sizes were tested using Levene’s test for equal variance.

We examined scaling relationships between moray size and bite force using standardized major axis (SMA) regressions in the R package smatr. Moray size measurements included the following morphometrics in mm: body mass, body length, head length, and head width. Scaling relationships were statistically compared using modified *t* tests with null predictions of the isometric slopes: 1.0 for linear measurements, 2.0 for areas and forces, and 3.0 for masses based on Euclidean geometry (Hill 1950; Schmidt-Nielsen 1984). We observed whether the predicted slopes fell within or outside the 95% confidence intervals of the observed SMA regression slopes as a guide for positive or negative allometry, respectively. We adjusted all *P* values using a Benjamini–Hochberg correction with an FDR *Q* value of 0.05 to reduce the type I error probability across multiple comparisons (Benjamini and Hochberg 1995). This same statistical protocol was used to test the hypothesis that prey size varied with bite force. We tested whether the relationship between prey size and moray head length or bite force was significantly different from our isometric predictions to determine whether larger bite force facilitates access to larger prey and whether larger morays were consuming larger prey. To do this, we used the prey size data extracted from the stomachs of individuals that closely matched the sizes of morays from when we measured in vivo bite force.

We then tested the prediction that morays exhibit an ontogenetic shift in average prey size. Since morays exhibit morphological adaptations for consuming large prey, we wanted to establish the range of maximum and minimum prey sizes across moray ontogeny. To do this, we adopted an approach by King (2002) which further uses regressions to examine the upper and lower bounds of prey. We first used ordinary least squares regression (OLS) of log-transformed moray head length and log-transformed prey standard length to determine the relationship between the two variables. Then, we analyzed the variation in data surrounding the regression by examining only positive and then only negative residuals. We ran subsequent OLS regressions for data points falling above the regression line (points with positive residuals) and those data below the regression line (points with negative residuals) to determine whether the lines forming the upper and lower bounds of prey size were significantly different from a slope of 0. Slopes that were significantly different from 0 suggest that maximum and minimum prey size increases with moray size.

## Results

### Gut contents

Between 2012 and 2016, we trapped 1338 moray eels across our six trapping sites (Fig. 1). From these morays, we isolated 169 distinguishable dietary items from the stomachs of 196 *G. mordax* (14.6%). The proportion of trapped morays with food in their stomachs varied little from 2013 to 2016 (16–17%). Ironically, in 2012, we trapped the most morays, but retrieved the least amount of dietary items (8%).

**Fig. 3:**
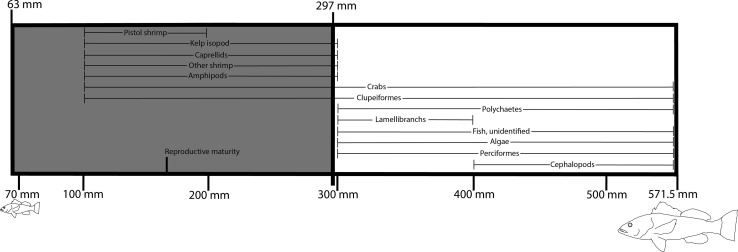
Size range of kelp bass consumed (*N* = 81) by *G. mordax* (shaded area) relative to reported size range of the species. Total size range of kelp bass was from data reported in Young, 1963. The reproductive maturity (178 mm *L*_T_) and most frequently consumed dietary items of kelp bass relative to size are overlaid (data from Quast, 1968)

During our 5 years of trapping, ~ 72% of the morays with stomach contents contained only a single dietary item in their gut. The most we recovered from a single moray was four kelp bass (*Paralabrax clathratus*). Moray diet consisted mainly of fish (range: 69–95%), with kelp bass as the most frequently consumed prey item (range: 63–297 mm, *L*_T_; Fig. 3). On average, kelp bass composed ~ 64% of the dietary items recovered (range over the years: 40–93%). ANOVA revealed that the average size of consumed kelp bass varied across years (*p *<0.0001; *df* = 4; *F* = 12.75). A Tukey’s post hoc test showed that the average size of kelp bass that morays consumed in 2015 (103 mm) was significantly smaller than those consumed in 2014 (140 mm; *p *<0.0001), 2016 (135 mm; *p *< 0.05), and 2012 (172 mm; *p *< 0.0001). However, mean kelp bass size consumed in 2015 and 2013 was not significantly different (*p *> 0.37).

The second and third most frequently consumed items were red rock shrimp (*Lysmata californica)* and two-spotted octopus (*Octopus bimaculoides*), respectively (Supplementary Table 1). Harder prey items such as kelp crab (*Pugettia productus*) and California spiny lobster (*Panulirus interruptus*) were also retrieved from the stomachs; however, these items were typically on the smaller end of the size range for the species (< 100 mm) and infrequently consumed (8 times over 5 years; ~ 5% of all dietary items). Two mantis shrimp (*Hemisquilla ensigera*) were recovered from the stomachs of morays (in 2013 and 2016), and a single blind goby (*Typhlogobius californiensis*) was recorded in 2015. Other notable dietary items include juvenile garibaldi (*Hypsypops rubicundus*), blacksmith (*Chromis punctipinnis*), and conspecifics; cannibalism was only observed in 2015 and 2016 (Table S1). The number of prey items consumed did not vary across years (Kolmogrov–Smirnov test, 0.09 < *p *< 0.7). Similarly, no significant difference was detected between the proportions of different types of prey items (kelp bass, mixed fishes, crustaceans, and molluscs) consumed across years (Kolmogrov–Smirnov test, 0.09 < *p* < 0.94).

**Fig. 4:**
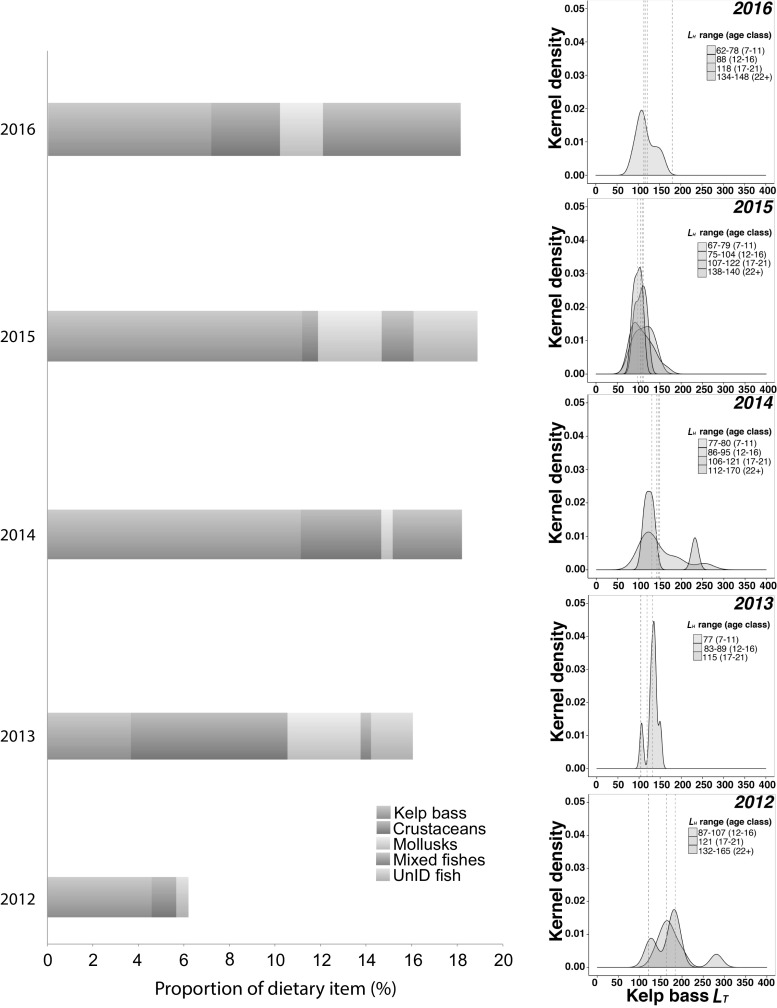
Bars represent the contribution of five prey categories to the diet of *G. mordax*. Each bar reflects diet data for a single year. Kernel density plots are displayed on the right with mean size (vertical lines) of kelp bass consumption by *L*_H_ range (mm) and age class, in parentheses. *L*_H_ were converted to age class date following the regression line presented in Higgins et al. (2017)

We binned morays into four-size categories and looked for patterns between moray size categories and the known size range of kelp bass consumed across years (Fig. 4). While at least one individual from all size categories was represented across the 5 years, the most common size category of moray for which we removed stomach contents was the 75–106 mm *L*_H_ category. In 2015, stomach contents from multiple individuals from all four-size categories were represented.

### Dietary habits: specialist or generalist

**Fig. 5:**
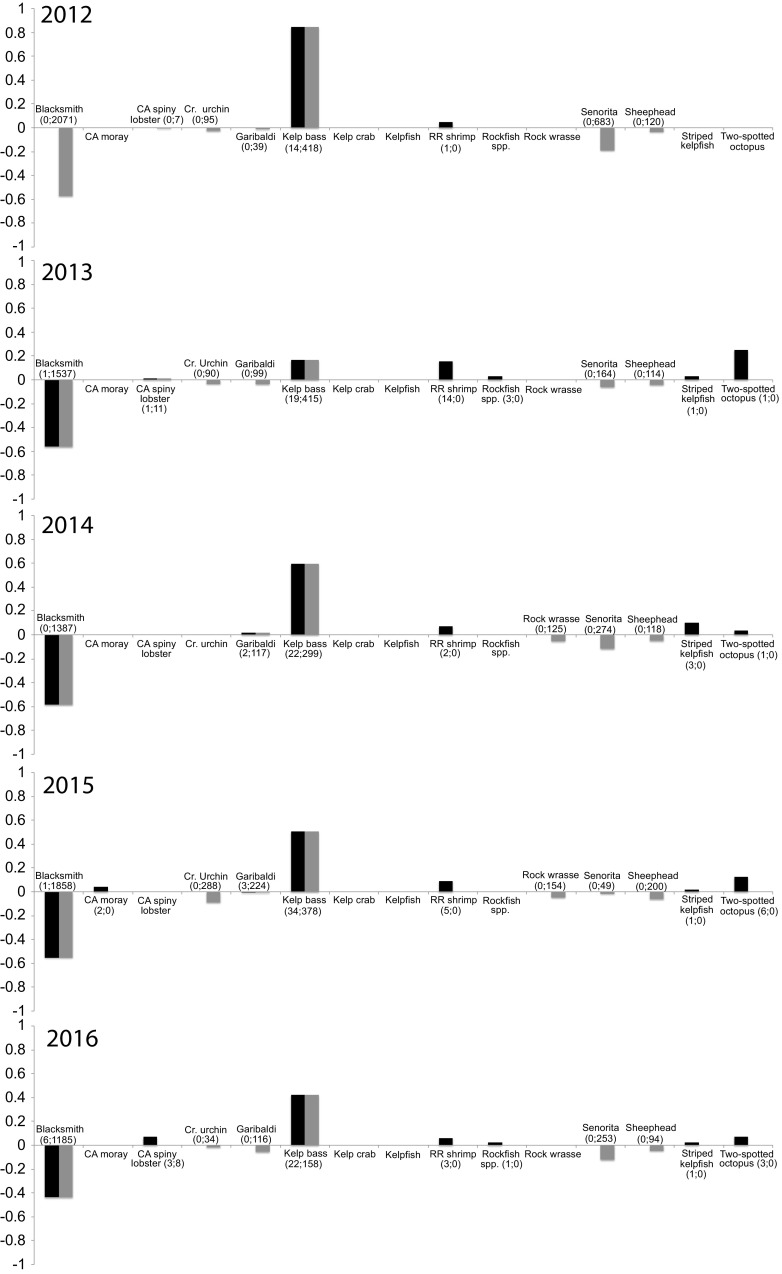
Paired linear selectivity index (*L*) values for the dietary items *G. mordax* consumed across years (black bars). Grey bars indicate *L* values for those items counted (if present) by Reef Check California survey transects. Numbers in parentheses indicate number of items morays consumed; number of individuals Reef Check California counted

Although linear selectivity index values (*L*) varied across years (Fig. 5), kelp bass consistently exhibited the highest *L* relative to other items in the habitat in all years except 2013 *L* range = 0.84–0.17; *L* in 2013 = 0.17). Two-spotted octopus exhibited the highest *L* in 2013 (0.25). Red rock shrimp *L* range = 0.04 (2012)–0.18 (2013) and two-spotted octopuses *L* range = 0.03 (2014)–0.25 (2013) were the only other dietary items that displayed consistent positive *L* values across years. We found that morays did not consume the most commonly occurring species in the environment. Although blacksmith was the most dominant vertebrate species counted in transect surveys, these fish were infrequent in the stomachs of morays (*L* range = − 0.50 to − 0.44). Señorita (*Oxyjulis californica*) and California sheephead (*Semicossyphus pulcher*) were also commonly observed in the environment, but neither of these species were ever recovered from moray stomachs. Overall, these results suggest that *G. mordax* primarily specializes on kelp bass with invertebrates serving as supplementary prey.

### Bite force

**Fig. 6:**
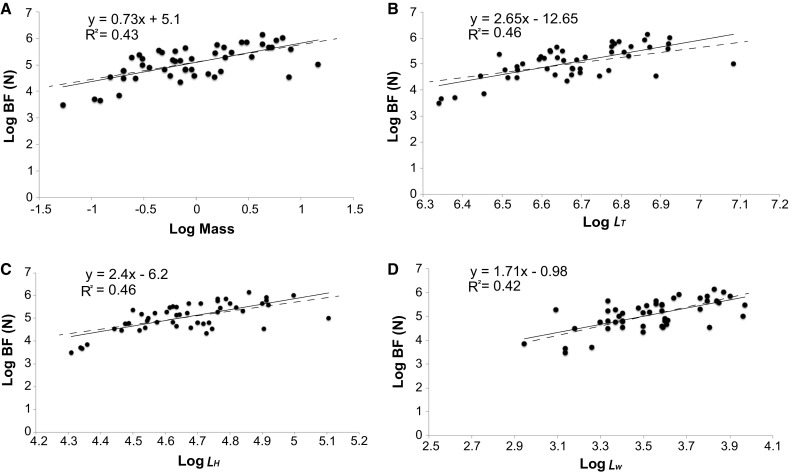
Relationships between *G. mordax* bite force (log) and mass (**a**), total length (**b**), head length (**c**), and head width (**d**). All variables, with the exception of head width, display a positive allometric relationship with bite force (*R*^2^ range: 0.42–0.46). Head width exhibited a negative allometric relationship. Dashed lines represent an isometric slope

We collected bite force measurements on 49 *G. mordax* (range: 567–1192 mm *L*_T_; mean: 804 mm *L*_T_) during the 2015 and 2016 trapping seasons. Bite forces ranged from 32.69 to 467.69 N and scaled with strong positive allometry with most morphological measurements (mass: *R*^2^ = 0.43, slope = 0.73; *L*_T:_*R*^2^ = 0.46, slope = 2.65); and *L*_H_:*R*^2^ = 0.46, slope = 2.4; both *p* < 0.0001). Head width (*L*_w_) was the only morphological feature that showed a negative allometric relationship with bite force (*R*^2^ = 0.42, slope = 1.71, *p *< 0.0001; Fig. 6). Based on the strong allometry between bite force and head length, we tested the relationship between prey size (*SL*_mm_) and bite force. We found no relationship between prey size and bite force in each of our prey categories (kelp bass, *p *> 0.383); mixed fishes, *p *> 0.684; and invertebrates, *p *> 0.665), suggesting that an increase in bite force does not facilitate morays consuming larger kelp bass, larger mixed fishes, or invertebrate prey.

### Predator–prey size relationships

We measured total lengths (*L*_T_) for 125 wholly intact prey items. The largest dietary item recovered was a kelp bass (297 mm, *L*_T_), which was consumed by the largest moray in our data set (1195 mm, *L*_T_). This kelp bass length was ~ 26% of the moray’s total length and ~ 169% of its *L*_H_.

**Fig. 7:**
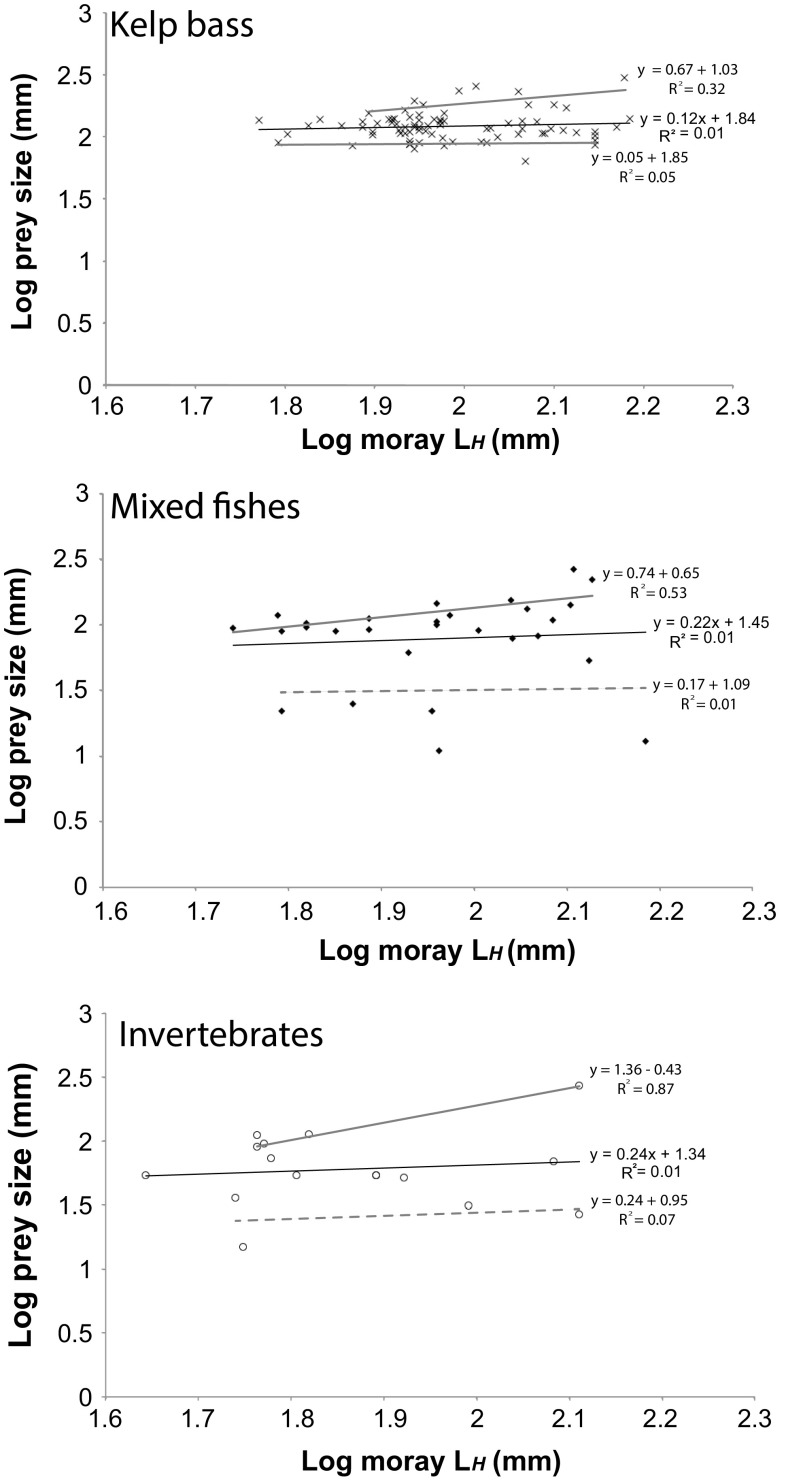
Degrees of ontogenetic shift (kelp bass) and telescoping (mixed fishes and invertebrates) using the relationship between *G. mordax* head length (log) and prey size (log) for kelp bass (*N* = 81), mixed fishes (*N* = 28), and invertebrate prey (*N* = 16). Black lines represent the OLS regression through the entire data set for each prey category. Thick grey lines represent OLS regression through positive and negative residual points. In all prey categories, we observe that the slope of the relationship between prey size and head length is significantly different from 0. Solid grey lines indicate slopes significantly greater than 0 for prey in the maximum size category, whereas dashed grey lines indicate slopes not significantly different from 0. For kelp bass prey, larger morays dropped smaller prey items from their diet that reveals an ontogenetic shift in diet, as opposed to ontogenetic telescoping observed in mixed fishes and invertebrates

The smallest prey consumed was a kelp crab (11 mm, carapace length), which was extricated from a moray measuring 692 mm in *L*_T_ (1.6% of the moray’s *L*_T_). There was no relationship between moray *L*_H_ and prey length for all prey categories (kelp bass: *p *>0.372; mixed fishes: *p *>0.644; invertebrate prey: *p *> 0.665). However, the regression lines between moray *L*_H_ and maximum and minimum prey size for kelp bass significantly differed from 0 (maximum and maximum, *p *<0.001), indicating an ontogenetic shift, where maximum and minimum sizes of kelp bass increased throughout ontogeny (Fig. 7). The regression lines for moray *L*_H_ and maximum size for mixed fishes and invertebrates also indicated slopes significantly greater than 0 (slopes = 0.12–0.24; *p *< 0.001). Maximum invertebrate size was retested without the apparent outlier and still returned a slope significantly different from 0 (*p *< 0.001). Therefore, with invertebrate prey less common in the moray diet, this outlier remained in the data set. Slopes for moray *L*_H_ and minimum size for mixed fishes (*p *=1) and invertebrates did not differ significantly from 0 (*p *= 1). These results reveal an ontogenetic telescoping pattern for mixed fishes and invertebrate prey, but where the minimum prey size does not increase over ontogeny. This suggests that while larger morays do eat larger fishes from a variety of taxa and larger invertebrates, the smallest of these prey do not drop out of their diet as observed with kelp bass prey.

## Discussion

### Functional ecology of *G. mordax*

We provide the first detailed multi-year data set on diet for *G. mordax*, showing that in Two Harbors, Catalina Island, the California moray is a piscivorous predator that specializes on kelp bass. Furthermore, we found a clear pattern of ontogenetic shift for kelp bass, where maximum prey size increases with moray size, but small prey are dropped from the diet of larger individuals (King 2002; Arnold 1993). However, the inclusion of secondary and typically smaller prey items in the stomach such as mantis shrimp, spiny lobster, blind gobies, and red rock shrimp suggests that morays are also opportunistic in their feeding behaviors. Supporting this idea is our finding that crustaceans were the most consumed prey item in 2013 when kelp bass were not abundant.

The inclusion of small mixed fishes and crustaceans in the stomachs of even the largest morays also supports the idea that individuals may be somewhat opportunistic about prey and their relative sizes. The previous studies on predator–prey dynamics in fishes reveal that, contrary to the predictions of optimal foraging models (see Ivlev 1961; Harper and Blake 1988), patterns of prey size consumption by predators do in fact include the retention of smaller prey despite larger predator sizes (Juanes and Conover 1995; Scharf et al. 2000). One hypothesis for why larger fish predators continue to consume small prey is that the importance of size-dependent capture success and differential encounter probabilities outweighs that of handling time (Scharf et al. 2000). Moreover, the abundance of small prey within the system may be significantly greater than those of larger prey, further elevating the likelihood of encounters between large predators and small prey (Scharf et al. 2000). This may in part help explain why we observed significantly smaller kelp bass sizes consumed in 2015 relative to all other years, where they had the largest *L* value. During the entirety of 2015, Santa Catalina Island was enveloped by the strongest El Niño since 1983 (Higgins et al. 2017). This resulted in a complete loss of kelp canopy cover (B.H., C.L. and R.M., pers. obs.) that kelp bass use for refuge during daytime hours (Ebeling and Bray 1976). Thus, kelp bass recruits that typically took refuge in the water column likely had to hide within the reef, thereby increasing the encounter rates between small kelp bass and morays. In years, where smaller kelp bass may not be as abundant, morays could function as stabilizing predators by opportunistically consuming a wide range of prey in a frequency-dependent regime, as we observed with the increasing, although not significant, proportion of invertebrate prey in the moray’s diet in 2013. These findings have strong ecological significance for the community as a whole, because a functional specialist such as *G. mordax,* provides a mechanism for maintaining elevated biodiversity through compensatory mortality (Connell 1978).

### Predator and prey size shifts across years

While the proportion of prey in the diet of the California moray did not vary significantly across years, we found annual differences in the average size of kelp basses consumed. The average size of kelp bass consumed was smallest in 2015, whereas the average size of kelp bass consumed was largest in 2012. These averages are just below the reproductive size of kelp bass (Fig. 3). Over the course of the 5-year study, we observed that moray predators of a wide size range were consuming kelp bass (Fig. 4). Despite the size variation in morays, there was strong overlap in kelp bass size. In our previous study, the distribution and abundance of moray sizes were uneven across different coves within Two Harbors, Catalina Island. The largest morays were trapped only in coves with east/northeastern-oriented faces (Higgins and Mehta 2017). Therefore, morays originating from different trapping sites that displayed different size structuring would all be consuming kelp bass prey of similar sizes. In addition, size-based predation frequencies across years may have been determined by fluctuations in reproductive output of kelp bass which would then lead to varying strengths of kelp bass recruitment pulses.

### Bite force and predation pressure

Within fishes that utilize biting as the primary mechanism to capture and consume prey, the size of the gape is often the factor that limits the types and sizes of prey that can be exploited (Kardong 2014). Constraints in cranial growth and/or morphological adaptations, however, often limit the biting ability and, therefore, may prohibit access to different prey species or prey items of a particular size (Herrel et al. 2006; Bulté et al. 2008; Santana et al. 2010; Pfaller et al. 2011). In this study, we found that morays exhibited a relatively high range of bite forces for their size especially when compared to other apex or secondary predators. For example, in situ bite force of the sympatric and almost exclusively durophagus horn shark (*Heterodontus francisci*) at a mass of 2.95 kg was 160 N (Huber et al. 2005). This is comparable to our in vivo bite forces recorded for a moray measuring 2.46 kg (266.54 N). In addition, moray bite forces increased disproportionately as head and body increased in size, suggesting that allometric increases in bite forces may enable the oral jaws to retain larger fish prey during feeding bouts or even provide accessibility to hard shelled prey.

Despite exhibiting allometrically increasing bite forces, our dietary data did not support ontogenetic shifts across all dietary items for morays. For example, morays did not transition from a piscivorous diet to a more durophagous diet (or vice versa) with increasing bite forces, but instead, fed on prey items proportional to moray head length throughout ontogeny. This hypothesis supports the previous findings that moray jaw dentition exhibited predominantly isometric growth, suggesting that the oral teeth grow proportionately as individuals increase in size (Harrison et al. 2017). Tooth growth patterns help explain why similar sized items are consumed by morays that vary widely in size as was found in the current study. Fracture forces for hard prey such as lobster and kelp crab are necessary to test the idea that allometric increases in bite forces may enable morays to consume a wider variety (type or size) of hard shelled prey when kelp bass recruits are not abundant.

Based on the strong allometric pattern of bite force, we would expect larger morays to consume fishes that exceed their head lengths. However, we found no significant relationship between prey size and moray bite force. Our results, therefore, suggest that kelp bass size selection is not limited by moray bite force. Rather, other variables such as encounter rates, capture rates, or handling times could limit the sizes of kelp bass prey in the California moray’s diet. California morays, similar to other morays (Miller 1987, 1989) or eel species (Helfman and Clark 1986), are known to ram, shake, knot, or use body rotations, to force large prey into their mouths or to remove pieces from larger prey items. In a previous study, we showed that prey size increased total feeding time and prey manipulation duration when morays were fed dead fish or cephalopod prey (Diluzio et al. 2017). Feeding durations and energetic demands necessary to capture and handle large live prey of increasing size would undoubtedly affect the caloric benefits of going after these larger prey.

### Frequency of predation

Of the morays trapped over the 5-year period, we found that the overwhelming majority had empty stomachs (85.4%). The previous studies have shown that piscivores have empty stomachs more often than non-piscivores and that nocturnal fishes tend to run empty more often than diurnal fishes. Piscivorous fishes that consume prey whole also tended to have the highest proportions of empty stomachs (Arrington et al. 2002). Our data set revealed that the California moray, a nocturnal piscivore, infrequently turned up with stomach contents averaging ~ 14.6% over the 5-year period in summer months. This low percentage of stomach contents could reflect the challenge of capturing kelp bass prey as piscivores tend to be less successful compared to planktivores (Juanes et al. 2002). Capture success in piscivores has also been shown to decrease when prey size to predator size ratio increases (Miller et al. 1988). The challenge of capturing fish prey has leaded others to speculate that maximum fishes consumed by piscivores are often considerably smaller than what would be predicted by predator gape size alone (Juanes and Conover 1995; Christensen 1996). Therefore, while predator gape size or cleithrum width may biomechanically limit the sizes of prey a predator may ingest, the behavioral abilities (i.e., evasiveness) of the prey may in fact more tightly regulate the sizes and types of prey consumed before gape morphology of the predator interacts with feeding. Alternatively, California morays may have low metabolic rates and individuals may not need to consume prey frequently. Higgins and Mehta (2017) showed that the body condition of morays was relatively consistent across moray size categories and coves suggesting if preferred prey are challenging to capture, it is not reflected in moray body condition.

### Trophic placement

Evidence from studies conducted in the tropics suggests that morays are predatory fishes that can be found in densities similar to commercially important predatory fishes such as serranids (sea basses and groupers) and lutjanids (snappers) (Gilbert et al. 2005) and can alter future community structure by preying upon newly settled recruiting fishes on Caribbean patch reefs (Parrish et al. 1986; Carr and Hixon 1995; Young and Winn 2003). Within the coves of Two Harbors, *G. mordax* is an abundant, static, carnivorous predator (Higgins and Mehta 2017; Harrison et al. 2017) that specializes on kelp bass, but can consume a relatively wide diversity of prey species throughout ontogeny. These results, therefore, suggest that morays have the densities to inflict consistent and elevated predation pressures on their prey populations as tertiary consumers; however, metabolic data would be necessary to understand the effects of *G. mordax* on various prey populations. The California moray was previously categorized as a secondary consumer (Graham 2004). Under this classification, *G. mordax* at two harbors is grouped in the same carnivorous fish category as their primary prey, kelp bass. Our results, suggest that *G. mordax* should be positioned above the carnivorous fishes category and is a tertiary consumer alongside sharks, rays, pinnipeds, and birds. While morays are apex consumers in tropical waters (Carr and Hixon 1995; Page et al. 2013), we hesitate to label *G. mordax* as an apex consumer without additional field observations on the habits of other resident marine predators, such as Harbor seals, *Phoca vitulina.*

## Electronic supplementary material

Below is the link to the electronic supplementary material.

Supplementary material 1 (PDF 297 kb)

## References

[CR1] Allen LG, Findlay AM, Phalen CM (2002). Structure and standing stock of the fish assemblages of Sand Diego Bay, California from 1994 to 1999. Bull South Calif Acad Sci.

[CR2] Anderson RA, McBrayer LD, Herrel A (2008). Bite force in vertebrates: opportunities and caveats for use of a nonpareil whole-animal performance measure. Biol J Lin Soc.

[CR3] Andersson M, Erlinge S (1977). Influence of predation on rodent populations. Oikos.

[CR4] Arnold SJ (1983). Morphology, performance and fitness. Integr Comp Biol.

[CR5] Arnold SJ, Collins JT (1993). Foraging theory and prey size–predator size relations in snakes. Siegel RA.

[CR6] Arrington DA, Winemiller KO, Loftus WF, Akin S (2002). How often do fishes “run on empty”?. Ecology.

[CR8] Benjamini Y, Hochberg Y (1995). Controlling the false discovery rate: a practical and powerful approach to multiple testing. J R Stat Soc Ser B.

[CR9] Bulté G, Irschick DJ, Blouin-Demers G (2008). The reproductive role hypothesis explains trophic morphology dimorphism in the northern map turtle. Funct Ecol.

[CR11] Carr M, Hixon M (1995). Predation effects on early post-settlement survivorship of coral-reef fishes. Mar Ecol Prog Ser.

[CR12] Christensen B (1996). Predator foraging capabilities and prey antipredator behaviours: pre- versus postcapture constraints on size-dependent predator–prey interactions. Oikos.

[CR13] Connell JH (1978). Diversity in tropical rain forests and coral reefs. Science.

[CR14] Cowen RK (1985). Large scale pattern of recruitment by the labrid, *Semicossphyus pulcher*: causes and implications. J Mar Res.

[CR17] de Roos AM, Persson L (2013). Poluation and community ecology of ontogenetic development.

[CR18] Diluzio AR, Baliga VB, Higgins BA, Mehta RS (2017). Effects of prey characteristics on the feeding behaviors of an apex marine predator, the California moray (*Gymnothorax mordax*). Zoology.

[CR19] Ebeling A, Bray RN (1976). Day versus night activity of reef fishes in a kelp forest off Santa Barbara, California. Fish Bull US.

[CR20] Erickson GM, Lappin AK, Vliet KA (2003). The ontogeny of bite-force performance in American alligator (*Alligator mississippiensis*). J Zool.

[CR21] Fitch HS, Scott NJ (1987). Resources of a snake community in prairie-woodland habitat of northeastern Kansas. Herpetological communities: a symposium of the Society for the Study of Amphibians and Reptiles and the Herpetologists’ League, August 1977.

[CR22] Froeschke JT, Allen LG, Pondella DJ (2006). The fish assemblages inside and outside of a temperate marine reserve in Southern California. Bull South Calif Acad Sci.

[CR23] Gilbert M, Rasmussen JB, Kramer DL (2005). Estimating the density and biomass of moray eels (Muraenidae) using a modified visual census method for hole dwelling reef fauna. Environ Biol Fishes.

[CR24] Graham MH (2004). Effects of local deforestation on the diversity and structure of Southern California giant kelp forest food webs. Ecosystems.

[CR25] Harper DG, Blake RW (1988). Energetics of piscivorous predator–prey interactions. J Theor Biol.

[CR26] Harrison JS, Higgins BA, Mehta RS (2017). Scaling of dentition and prey size in the California moray (*Gymnothorax mordax*). Zoology.

[CR80] Helfman GS, Clark JB (1986). Rotational feeding: overcoming gape-limited foraging in anguillid eels. Copeia.

[CR27] Herrel A, Joachim R, Vanhooydonck B, Irschick DJ (2006). Ecological consequences of ontogenetic changes in head shape and bite performance in the Jamaican lizard *Anolis lineatopus*. Biol J Lin Soc.

[CR28] Higgins BA, Mehta RS (2017). Distribution and habitat associations of the California moray (*Gymnothorax mordax*) within Two Harbors, Santa Catalina Island, California. Environ Biol Fishes.

[CR29] Higgins BA, Pearson D, Mehta RS (2017). El Niño episodes coincide with California moray *Gymnothorax mordax* settlement around Santa Catalina Island, California. J Fish Biol.

[CR30] Hill AV (1950). The dimensions of animals and their muscular dynamics. Sci Prog.

[CR31] Huber DR, Eason TG, Hueter RE, Motta PJ (2005). Analysis of the bite force and mechanical design of the feeding mechanism of the durophagous horn shark *Heterodontus francisci*. J Exp Biol.

[CR32] Ivlev VS (1961). Experimental ecology of the feeding of fishes.

[CR33] Jacobson P, Gårdmark A, Östergen J, Casini M, Huss M (2018). Size-dependent prey availability affects diet and performance of predatory fish at sea: a case study of Atlantic salmon. Ecosphere.

[CR34] Juanes F, Stouder DJ, Fresh KL, Feller RJ (1994). What determines prey size selectivity in piscivorous fishes?. Theory and application in fish feeding ecology.

[CR73] Juanes F, Buckel JA, Scharf FS (2002) Feeding ecology of piscivorous fishes. In: Hart PJB, Reynolds JD (eds) Handbook of fish biology and fisheries: fish biology. Wiley

[CR35] Juanes F, Conover DO (1995). Size-structured piscivory: advection and the linkage between predator and prey recruitment in young-of-the-year bluefish. Mar Ecol Prog Ser.

[CR37] Kardong KV (2014). Vertebrates: comparative anatomy, function, evolution.

[CR38] King RB (2002). Predicted and observed maximum prey size–snake size allometry. Funct Ecol.

[CR39] Lappin AK, Jones ME (2014). Reliable quantification of bite-force performance requires use of appropriate biting substrate and standardizing of bite out-lever. J Exp Biol.

[CR41] Marshall CD, Guzman A, Narazaki T, Katsufumi S, Kane EA, Sterba-Boatwright BD (2012). The ontogenetic scaling of bite force and head size in loggerhead sea turtles (*Caretta caretta*): implications for durophagy in neritic, benthic habitats. J Exp Biol.

[CR42] Mehta RS, Wainwright PC (2007). Biting releases constraints on moray eel feeding kinematics. J Exp Biol.

[CR43] Miller T (1987). Knotting: a previously undescribed feeding behavior in muraenid eels. Copeia.

[CR44] Miller T (1989). Feeding behavior of *Echidna nebulosa, Enchelycore pardalis,* and *Gymnomuraena zebra* (Teleostei: Muraenidae). Copeia.

[CR45] Miller TJ, Crowder LB, Rice JA, Marschall EA (1988). Larval size and recruitment mechanisms in fishes: toward a conceptual framework. Can J Fish Aquat Sci.

[CR46] Mittelbach GG (1981). Foracing efficiency and body size: a study of optimal diet and habitat use by bluegills. Ecology.

[CR47] Mittelbach GG, Persson L (1998). The ontogeny of piscivory and its ecological consequences. Can J Fish Aquat Sci.

[CR48] Morin PJ (2011). Community ecology.

[CR49] Murdoch WW (1969). Switching in general predators: experiments on predator specificity and stability of prey populations. Ecol Monogr.

[CR50] Mushinsky HR, Hebrard JJ (1977). Food partitioning by fish species of water snakes in Louisiana. Herpetologica.

[CR51] Osenberg CW, Mittelbach GG (1989). Effects of body size on the predator–prey interaction between pumpkinseed sunfish and gastropods. Ecol Monogr.

[CR52] Page H, Brooks A, Kulbicki M, Galzin R (2013). Stable isotopes reveal trophic relationships and diet of consumers in temperate kelp forest and coral reef ecosystems. Oceanography.

[CR53] Parrish JD, Norris JE, Callahan MW, Callahan JK, MagarifujiI EJ, Schroeder RE (1986). Piscivory in a coral reef fish community. Environ Biol Fish.

[CR54] Pérez-Barbería FJ, Gordon IJ (1999). The functional relationship between feeding type and jaw and cranial morphology in ungulates. Oecologia.

[CR55] Persson L, Hughes RN (1990). Predicting ontogenetic niche shifts in the field: what can be gained by foraging theory?. Behavioural mechanisms of food selection.

[CR56] Persson L, Andersson J, Wahlstrom E, Eklov P (1996). Size specific interactions in lake systems: predator gape limitation and prey growth rate and mortality. Ecology.

[CR57] Pfaller JB, Gignac PM, Erickson GM (2011). Ontogenetic changes in jaw–muscle architecture facilitate durophagy in the turtle *Sternotherus minor*. J Exp Biol.

[CR81] Quast JC (1968) Observations on the food and biology of the kelp bass,* Paralabrax clathratus*. with notes on its sportfishery at San Diego, california. In: North WJ, Hubbs CL (eds) Utilization of kelp-bed resources in southern California, vol 139. California Department of Fish and Game, Fisheries Bulletin, pp 81–108

[CR58] Redpath SM, Thirgood SJ (1999). Numerical and functional responses in generalist predators: hen harriers and peregrines in Scottish grouse moors. J Anim Ecol.

[CR60] Santana SE, Dumont ER, Davis JL (2010). Mechanics of bite force production and its relationship to diet in bats. Funct Ecol.

[CR74] Sánchez-Hernandez J, Servia MJ, Vieira-Lanero R, Cobo F (2012) Ontogenetic dietary shifts in a predatory freshwater fish species: the brown trout as an example of a dynamic fish species. In: Tirker H (ed) Chapter 9 New advances and contributions to fish biology. InTech

[CR61] Scharf FS, Juanes F, Rountree RA (2000). Predator size–prey size relaionships of marine fish predators: interspecific variation and effects of ontogeny and body size on trophic-niche breadth. Mar Ecol Prog Ser.

[CR62] Schmidt-Nielsen K (1984). Scaling: why is animal size so important?.

[CR64] Shurin JB, Gruner DS, Hillebrand H (2006). All wet or dried up? Real differences between aquatic and terrestrial food webs. Proc R Soc B.

[CR65] Strauss RE (1979). Reliability estimates for Ivlev’s Electivity index, the forage ratio, and a proposed linear index of food selection. Trans Am Fish Soc.

[CR66] van der Meij MAA, Bout RG (2006). Seed husking time and maximal bite force in finches. J Exp Biol.

[CR67] Verwaijen D, Van Damme R, Herrel A (2002). Relationships between head size, bite force, prey handling efficiency and diet in two sympatric lacertid lizards. Funct Ecol.

[CR68] Wainwright PC, Richard BA (1995). Predicting patterns of prey use from morphology of fishes. Environ Biol Fishes.

[CR69] Wainwright PC, Shaw SS (1999). Morphological basis of kinematic diversity in feeding sunfishes. J Exp Biol.

[CR70] Werner EE (1977). Species packing and niche complementarity in three sunfishes. Am Nat.

[CR71] Woodward G, Ebenman B, Emmerson M, Montoya JM, Oleson JM, Valido A, Warren PH (2005). Body size in ecological networks. Trends Ecol Evol.

[CR82] Young PH (1963) The Kelp Bass (*Paralabrax clathratus*) and its fishery, 1947–1958. California Department of Fish and Game, Fisheries Bulletin 172

[CR72] Young R, Winn H (2003). Activity patterns, diet, and shelter site use for two species of moray eels, *Gymnothorax moringa* and *Gymnothorax vicinus*, in Belize. Copeia.

