# Supplementary material for: Eat whole and less often: ontogenetic shift reveals size specialization on kelp bass by the California moray eel, Gymnothorax mordax
Source: Oecologia. 2018 Sep 18;188(3):875–87. doi: 10.1007/s00442-018-4260-x (PMC6208710; doi:10.1007/s00442-018-4260-x)
Supplement: Supplementary file 1 — Supplementary material 1 (PDF 297 kb) [file 442_2018_4260_MOESM1_ESM.pdf]

Eat Whole and Less Often: Ontogenetic shift reveals size specialization on kelp bass by the California moray eel, *Gymnothorax mordax*

Higgins, Benjamin A*†, Law, Chris J. *, and Mehta, Rita S.*

*Department of Ecology and Evolutionary Biology, Center for Coastal Biology, 130 McAllister Way, Santa Cruz, CA 95060

† corresponding author: Ben Higgins; 130 McAllister Way, Santa Cruz, CA 95060; bahiggin@ucsc.edu;

RSM designed the study. RSM, BAH, and CJL collected the data. BAH analyzed the data. RSM, BAH, and CJL interpreted the data. BAH wrote the manuscript. RSM and CJL provided feedback on various iterations of the manuscript.

Supplemental methods

A series of underwater transects were placed in the summer of 2013 to examine the potential prey items found in the environment where morays are typically observed (Higgins & Mehta, 2017). We assessed prey distributions by depth, by running eight transects (four pairs of two) parallel to the shoreline at varying depths (3m, 8m, 13m, and 18m). Traps were set at each of these depths and two, 10m transects were laid parallel to the shoreline, resulting in a 20m transect for each trap at each depth. Two scuba divers swam along each arm of the transect recording all vertebrate and invertebrate species observed. As these transects (N = 32), were extremely time intensive to perform, we adopted the annual data monitoring transect surveys by Reef Check for years, 2012, 2014, 2015, and 2016. To determine whether our 2013 surveys could be comparable with Reef Check, we conducted a series of Z-tests to used to examine the differences in distributions of potential prey items detected in both Reef Check and our own surveys of prey items. Our statistical analyses did not detect a significant difference between the proportions of prey items quantified by the transects we conducted in 2013 and those conducted by Reef Check teams during the same year (Z-score range: $0.16 < p < 1$).

Supplemental Table 1: Dietary items recovered from the guts of 196 *G. mordax* via manual palpation.

Dietary item	2012	2013	2014	2015	2016
Blacksmith (*Chromis punctipinnis*)	-	1	-	1	2
Blind goby (*Typhlogobius californiensis*)	-	-	-	1	-
CA moray (*Gymnothorax mordax*)	-	-	-	2	2
CA spiny lobster (*Panulirus interruptus*)	-	-	-	-	3
Garibaldi (*Hypsypops rubicundus*)	-	-	2	1	-
Kelp bass (*Paralabrax clathratus*)	14	21	21	45	13
Kelp crab (*Pugettia producta*)	-	-	-	-	1
Mantis shrimp (*Hemisquilla ensigera*)	-	1	-	-	1
Octopus (*Octopus bimaculoides*)	-	1	1	5	2
Painted greenling (*Oxylebius pictus*)	-	-	-	-	1
Red rock shrimp (*Lysmata californica*)	1	5	2	1	1
Rockfish spp.	-	2	-	-	1
Sculpin spp.	-	1	-	-	-
Striped kelpfish (*Gibbonsia metzi*)	-	-	3	1	1
Unidentifiable crab	-	-	1	-	-
Unidentifiable fish	-	-	-	4	3
